# Supplementary figures and images for: Factors affecting integration of an early warning system for antimalarial drug resistance within a routine surveillance system in a pre-elimination setting in Sub-Saharan Africa
Source: PLoS One. 2025 Jun 3;20(6):e0305885. doi: 10.1371/journal.pone.0305885 (PMC12132925; doi:10.1371/journal.pone.0305885)

| 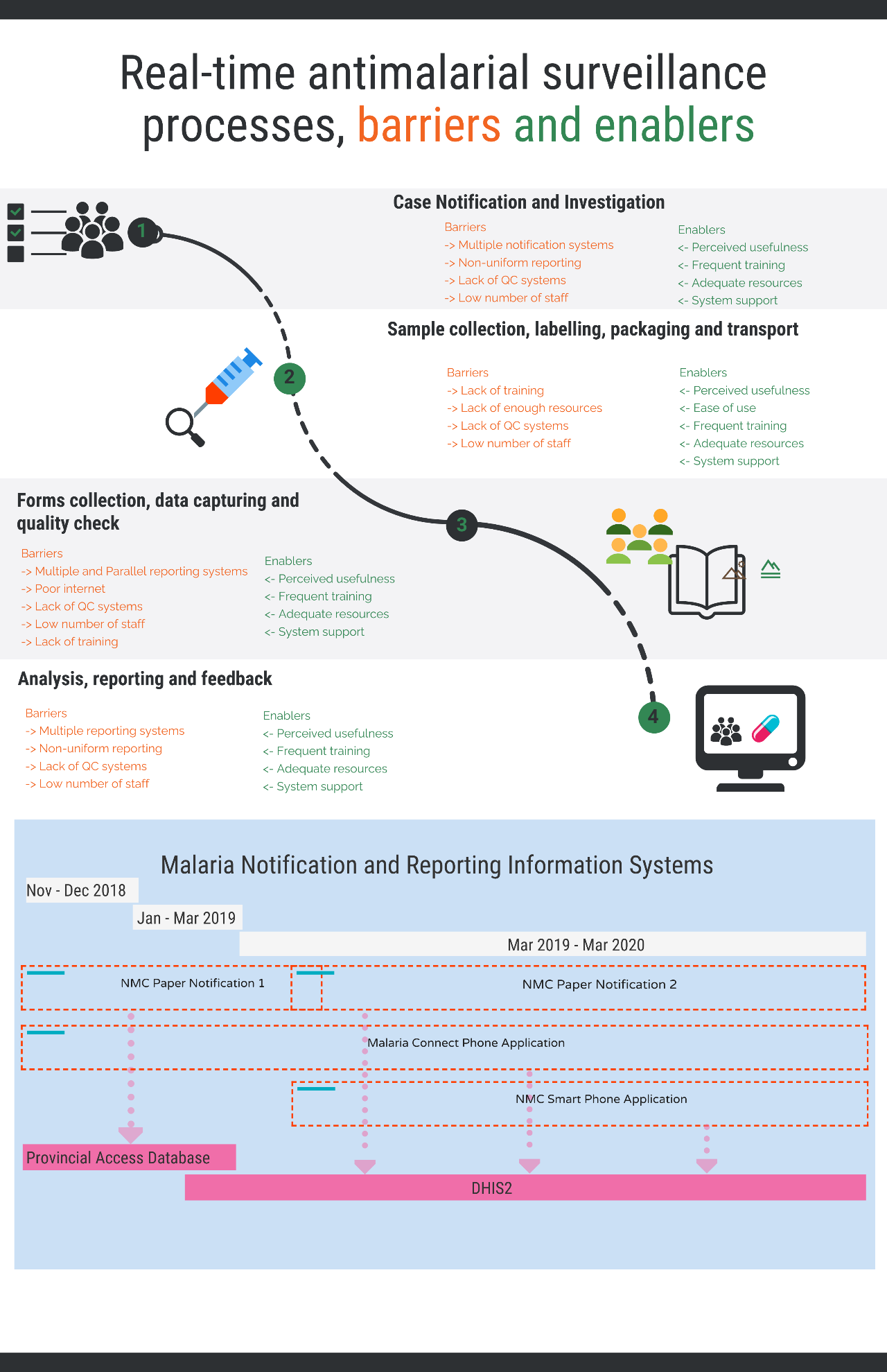 |
| --- |

Supplement: S1 Fig — (DOCX) [file pone.0305885.s001.docx]

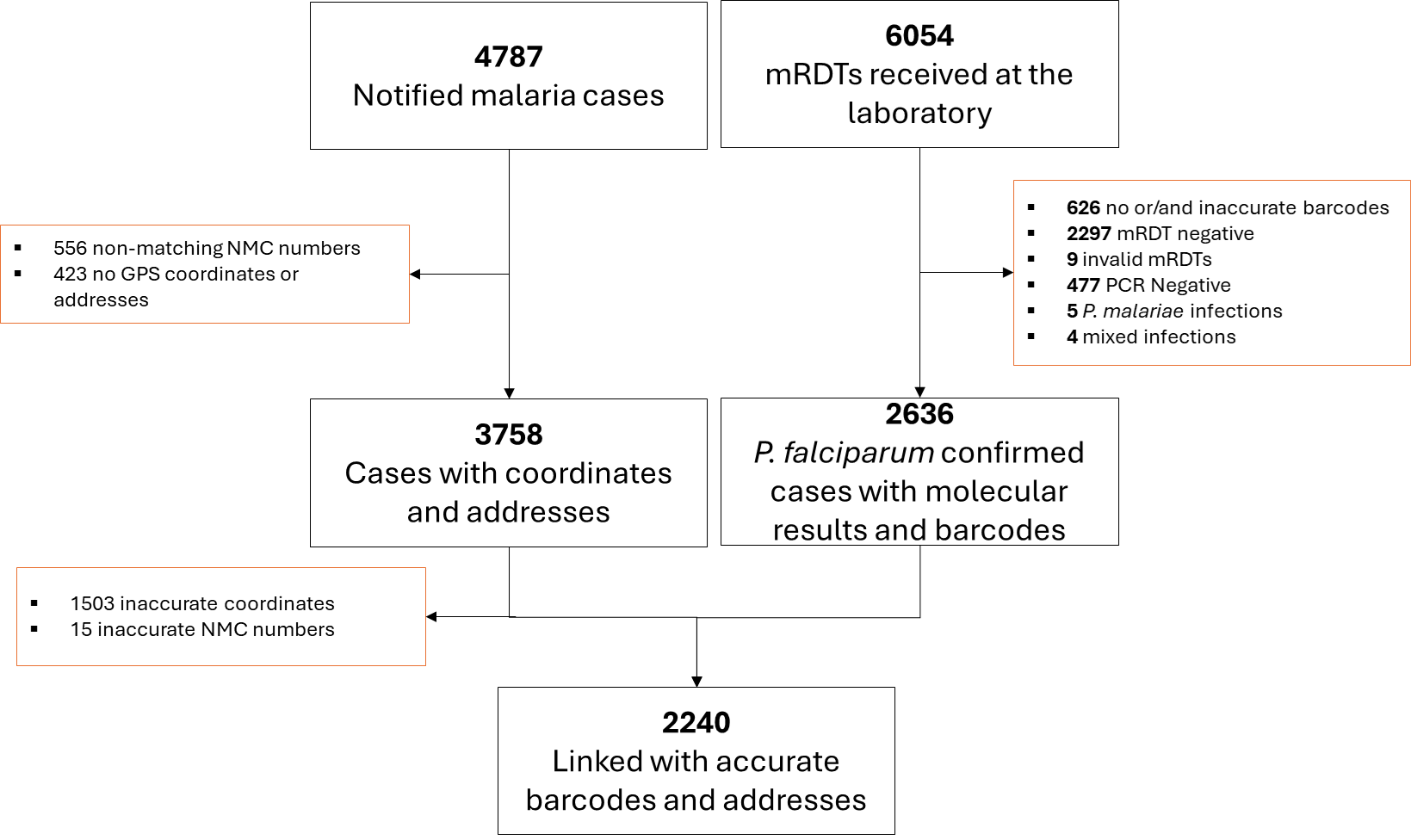

Supplement: S3 Fig — (DOCX) [file pone.0305885.s003.docx]
