# Supplementary material for: Factors affecting integration of an early warning system for antimalarial drug resistance within a routine surveillance system in a pre-elimination setting in Sub-Saharan Africa
Source: PLoS One. 2025 Jun 3;20(6):e0305885. doi: 10.1371/journal.pone.0305885 (PMC12132925; doi:10.1371/journal.pone.0305885)
Supplement: S2 Fig — Comparison of malaria case notifications (S2a) and investigations (S2b) from source (Health Care Facilities – HCF), DCC and DHIS2 using three HCFs as the primary source. Malaria case data were aggregated and compared in the different levels for every first month of the five quarters in Nkomazi, Mpumalanga South Africa. (DOCX) [file pone.0305885.s002.docx]

| 2(a) Malaria cases notified | |
| --- | --- |
| 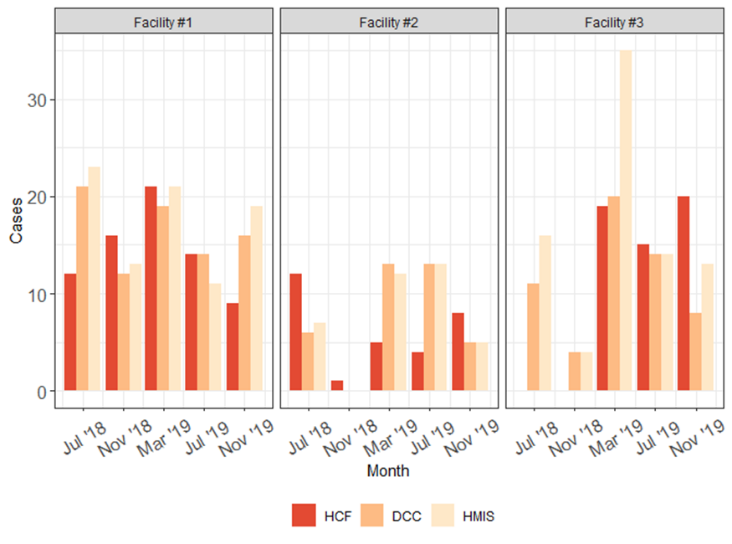 | In three HCFs, the case notification report counts matched in 40% (6/15) of the evaluations. Four of these six matching evaluations occurred in the latest two quarters (July and November 2019), which correlated with a period of training and supportive supervisory visits. At no quarter all three levels had similar counts. The two secondary levels had less data variability than their primary source health facility. |
| 2(b) Malaria Cases Investigated | |
| 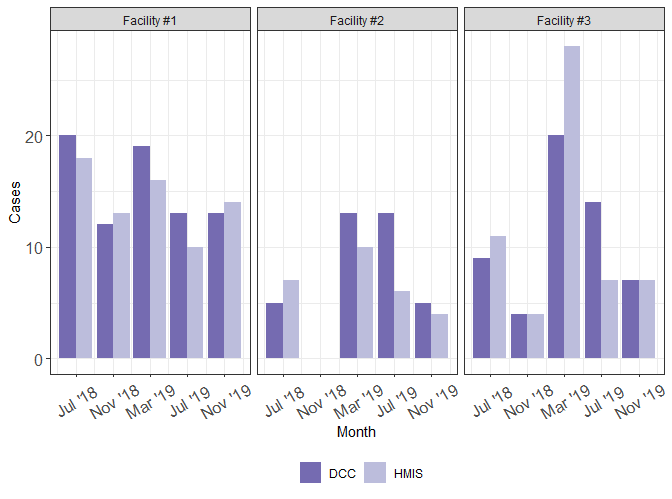 | Case investigation report counts matched across two levels in three evaluations (n=15) in September – November 2018 & 2019 and April – July 2019. The data between the DCC and DHIS2 differed in most evaluations (86.7%; 13/16), with the third level (HIMS) having more data than the second level in the remaining 3 evaluations. |
|  | |
