# Supplementary material for: Factors affecting integration of an early warning system for antimalarial drug resistance within a routine surveillance system in a pre-elimination setting in Sub-Saharan Africa
Source: PLoS One. 2025 Jun 3;20(6):e0305885. doi: 10.1371/journal.pone.0305885 (PMC12132925; doi:10.1371/journal.pone.0305885)
Supplement: S1 Tool — (DOCX) [file pone.0305885.s004.docx]

| **SS4ME project survey** | | | | | | |
| --- | --- | --- | --- | --- | --- | --- |
| **Clinic name** | |  | | **Clinic ID (if known)** |  | |
| **Your job title** | |  | | **Today’s date** | __/______/___  Dd/month/year | |
| **WE APPRECIATE YOUR HELP WITH THIS SURVEY ABOUT THE SS4ME PROJECT**  **THE QUESTIONS SHOULD TAKE ABOUT 30 MINUTES OF YOUR TIME**  **THERE ARE NO WRONG ANSWERS!** | | | | | | |
| **Please read this background to the survey first**  There are parts of the world where malaria parasites have developed resistance to all malaria treatments.  So far, the treatments recommended here work well, but we need to improve our systems to make sure we are able to detect and contain any resistance that occurs.  For the SS4ME project we collected positive and negative malaria RDTs from your clinic/hospital and sent them to a laboratory in Johannesburg (NICD). We also asked staff to collect and send a filter paper sample from any left-over blood instead of wiping it away with a webcol/cotton wool.  The lab is now testing the RDTs and blood spots to see if there was any resistance to Co-Artem® | | | | | | |
| **1** | **How was your experience of labelling individual RDTs?** | | **Mark ONE of the following 4 answers:** | | | |
|  |  |  | This is not part of my work  It is easy for me to do  It is sometimes difficult for me to do  It is difficult for me to do | | | 🞏  🞏  🞏  🞏 |
| **Please briefly explain your answer to question 1 here if you would like:** | | | | | | |
| **2** | **How was your experience of labelling individual filter paper samples (blood spots)?** | | **Mark ONE of the following 4 answers:** | | | |
|  |  | | This is not part of my work  It is easy for me to do  It is sometimes difficult for me to do  It is difficult for me to do | | | 🞏  🞏  🞏  🞏 |
| **Please briefly explain your answer to question 2 here if you would like:** | | | | | | |
| **3** | **How was your experience of putting positive RDTs into individual packets for collection by the malaria programme and negatives RDTs in a different bag?** | | **Mark ONE of the following 4 answers:** | | | |
|  |  |  | This is not part of my work  It is easy for me to do  It is sometimes difficult for me to do  It is difficult for me to do | | | 🞏  🞏  🞏  🞏 |
| **Please briefly explain your answer to question 3 here if you would like:** | | | | | | |
| **4** | **How was your experience of putting filter paper blood spots into packets for collection by the malaria programme?** | | **Mark ONE of the following 4 answers:** | | | |
|  |  |  | This is not part of my work  It is easy for me to do  It is sometimes difficult for me to do  It is difficult for me to do | | | 🞏  🞏  🞏  🞏 |
| **Please briefly explain your answer to question 4 here if you would like:** | | | | | | |
| **5** | **Which tools for malaria case notification have you used in the last 6 months?** | | **Check all that apply:** | | | |
|  |  |  | Malaria  Malaria Connect  NMC Phone app  Other (please name): ______________________________ | | | 🞏  🞏  🞏  🞏 |
| **6** | **How was your experience of using the paper NMC form to notify malaria cases?** | | **Mark ONE of the following 4 answers:** | | | |
|  |  |  | This is not part of my work  It is easy for me to use  It is sometimes difficult for me to use  It is difficult for me to use | | | 🞏  🞏  🞏  🞏 |
| **Please briefly explain your answer to question 5 here if you would like:** | | | | | | |
| **7** | **What was the effect of SS4ME on your work?** | | **Mark ONE of the following 3 answers:** | | | |
|  |  |  | It had no effect  It affected my work in a negative way (i.e. caused me problems with my work)  It affected my work in a positive way | | | 🞏  🞏  🞏 |
| **Please briefly explain your answer to question 7 here if you would like:** | | | | | | |
| **8** | **Do you think the SS4ME project is useful?** | | **Mark ONE of the following 3 answers:** | | | |
|  |  |  | Yes  No  Don’t know | | | 🞏  🞏  🞏 |
| **Please briefly explain your answer to question 8 here if you would like:** | | | | | | |
| **9** | **What should we do differently if we wanted to ask a new clinic to start SS4ME?** | | **Mark ONE of the following 2 answers:** | | | |
|  |  |  | Keep things as they are  Change things | | | 🞏  🞏 |
| **Please briefly explain your answer to question 9 here if you would like:** | | | | | | |
| **10** | **The department of health introduced a single low dose of primaquine to clinics in the last year. Please let us know how this has affected your work** | | **Mark ONE of the following 3 answers:** | | | |
|  |  |  | It had no effect  It affected my work in a negative way (i.e. caused me problems with my work)  It affected my work in a positive way | | | 🞏  🞏  🞏 |
| **Please briefly explain your answer to question 10 here if you would like:** | | | | | | |
| **Finally** | **Please write any other comments or suggestions that you can think of for improving malaria notifications in this area** **if you would like** | |  | | | |
| **THANK YOU FOR YOUR HELP** | | | | | | |
