# Supplementary material for: Factors affecting integration of an early warning system for antimalarial drug resistance within a routine surveillance system in a pre-elimination setting in Sub-Saharan Africa
Source: PLoS One. 2025 Jun 3;20(6):e0305885. doi: 10.1371/journal.pone.0305885 (PMC12132925; doi:10.1371/journal.pone.0305885)
Supplement: S2 Tool — (DOCX) [file pone.0305885.s005.docx]

| **Tools S2a** |
| --- |
| **INFORMATION AND CONSENT FORM – SURVEY**  **Smart surveillance towards malaria elimination in Mpumalanga, South Africa (SS4ME): novel approaches for mapping antimalarial resistance**  *Sub-study: Development and optimisation of Plasmodium falciparum artemisinin and partner drugs resistance maps for communication with policymakers in Southern Africa and South-East Asia* |
| **Introduction**  As you may know, we have been conducting a study to plan for a new approach for linking antimalarial drug resistance data with malaria notification data (Smart Surveillance for Malaria Elimination, SS4ME), which we piloted in Mpumalanga. We are interested in learning about how this project has been experienced from the perspective of those involved in malaria notification activities and those who would use the data generated to make policy decisions in this region. will also present some drafted maps obtained from the collected data and ask for your advice in ways we can improve them.  **Why was this study being done?**  We wanted to know how best activities related to SS4ME could be planned and integrated into the current malaria notification system. This involved the malaria elimination programme (MEP) collecting all positive RDT tests (with filter papers used to dab any blood remaining after the fingerprick) and sending them to the NICD laboratory for assays of molecular markers of resistance to Coartem®. These results were then linked to those of the malaria notifications and displayed on a map to indicate risk of antimalarial drug resistance and level of uncertainty. The results of this project will help us understand how best this SS4ME could be designed and implemented within malaria endemic areas of South Africa and potentially regionally.  **What will happen today if I take part in this study?**  Today, we would like to ask you questions about your perspective on the project we have now completed, including the value of linking these data with drug resistance marker data and how best this could be planned for and implemented now that we have completed the pilot; what worked, what didn’t work and how SS4ME may be improved. As before, we will take notes of the discussions and a recording will also be made using a digital voice recorder.  **What are my rights if I take part in this interview?**  Taking part in this interview is entirely your choice. You may choose either to take part or not to take part. If you decide to take part, you can decide to stop participating at any time. Just tell the project researcher right away if you wish to stop an interview. No matter what decision you take, there will be no penalty to you in any way.  **How long will the interview last and what risks can I expect from taking part?**  Each interview will last about 60-90 minutes. Participation in any research study may involve a loss of privacy. Information you provide about your experiences and opinions will be recorded, but your name will not be used in any reports of the information provided. No quotes or other results arising from your participation in this study will be included in any reports, even anonymously, without your agreement. The information obtained from these interviews will only be used by the project researchers and will be locked at our project offices. We will do our best to make sure that the personal information gathered for this survey is kept private.  **Are there benefits to taking part in the study?**  There will be no direct benefit to you from participating in this study. However, the information that you provide will help researchers and policy-makers understand how best to implement SS4ME.  **What are the costs of taking part in this study? Will I be paid for taking part in this study?**  There are no costs to you for taking part in this study. You will not be paid for taking part.  **Who can answer my questions about the study?**  You can talk to the researchers about any questions or concerns you have about this interview. If you have any questions, comments or concerns about taking part in this study, first talk to the researchers [Contact [name, number of project team member(s)]. If for any reason you do not wish to do this, or you still have concerns about doing so, you may contact the UCT Human Research Ethics Committee using the contacts below:  **HREC Ethics Office:**  **Email:** [**hrec-enquiries@uct.ac.za**](mailto:hrec-enquiries@uct.ac.za)  **Tel: +27 21 406 6338**  The study has been explained to me in a language that I understand. All the questions I had about the study have been answered. I understand what will happen during the interviews and what is expected of me.   - I have been informed that it is my right to refuse to take part in the interviews and that if I choose to refuse I do not have to give a reason, and that my employer will not be informed now, or in the future. - I have been informed that anything I say during the interviews will be kept confidential: my name will not be used and no quotes or other results arising from my participation in this study will be included in any reports, even anonymously, without my agreement.   Circle response:   \| I agree to take part in the study: \| Yes \| No \| \| --- \| --- \| --- \|   **Signature of participant:**   \| **NAME**  (in capital letters) \| **SIGNATURE** \| **DATE OF SIGNATURE**  (in DD/MM/YYYY) \| \| --- \| --- \| --- \| \|  \|  \|  \|   **Signature of study staff taking consent:**   \| **NAME**  (in capital letters) \| **SIGNATURE** \| **DATE OF SIGNATURE**  (in DD/MM/YYYY) \| \| --- \| --- \| --- \| \|  \|  \|  \| |

| **Tool S2b** |
| --- |
| **INFORMATION AND CONSENT FORM – SEMI-STRUCTURED INTERVIEWS (FGD)**  **Smart surveillance towards malaria elimination in Mpumalanga, South Africa (SS4ME): novel approaches for mapping antimalarial resistance**  *Sub-study: Development and optimisation of Plasmodium falciparum artemisinin and partner drugs resistance maps for communication with policymakers in Southern Africa and South-East Asia* |
| **Introduction**  As you may know, we have been conducting a study to plan for a new approach for linking antimalarial drug resistance data with malaria notification data (Smart Surveillance for Malaria Elimination, SS4ME), which we piloted in Mpumalanga. We are interested in learning about how this project has been experienced from the perspective of those involved in malaria notification activities and those who would use the data generated to make policy decisions in this region. will also present some drafted maps obtained from the collected data and ask for your advice in ways we can improve them.  **Why was this study being done?**  We wanted to know how best activities related to SS4ME could be planned and integrated into the current malaria notification system. This involved the malaria elimination programme (MEP) collecting all positive RDT tests (with filter papers used to dab any blood remaining after the fingerprick) and sending them to the NICD laboratory for assays of molecular markers of resistance to Coartem®. These results were then linked to those of the malaria notifications and displayed on a map to indicate risk of antimalarial drug resistance and level of uncertainty. The results of this project will help us understand how best this SS4ME could be designed and implemented within malaria endemic areas of South Africa and potentially regionally.  **What will happen today if I take part in this study?**  Today, we would like to ask you questions about your perspective on the project we have now completed, including the value of linking these data with drug resistance marker data and how best this could be planned for and implemented now that we have completed the pilot; what worked, what didn’t work and how SS4ME may be improved. As before, we will take notes of the discussions and a recording will also be made using a digital voice recorder.  **What are my rights if I take part in this interview?**  Taking part in this interview is entirely your choice. You may choose either to take part or not to take part. If you decide to take part, you can decide to stop participating at any time. Just tell the project researcher right away if you wish to stop an interview. No matter what decision you take, there will be no penalty to you in any way.  **How long will the interview last and what risks can I expect from taking part?**  Each interview will last about 60-90 minutes. Participation in any research study may involve a loss of privacy. Information you provide about your experiences and opinions will be recorded, but your name will not be used in any reports of the information provided. No quotes or other results arising from your participation in this study will be included in any reports, even anonymously, without your agreement. The information obtained from these interviews will only be used by the project researchers and will be locked at our project offices. We will do our best to make sure that the personal information gathered for this survey is kept private.  **Are there benefits to taking part in the study?**  There will be no direct benefit to you from participating in this study. However, the information that you provide will help researchers and policy-makers understand how best to implement SS4ME.  **What are the costs of taking part in this study? Will I be paid for taking part in this study?**  There are no costs to you for taking part in this study. You will not be paid for taking part.  **Who can answer my questions about the study?**  You can talk to the researchers about any questions or concerns you have about this interview. If you have any questions, comments or concerns about taking part in this study, first talk to the researchers [Contact [name, number of project team member(s)]. If for any reason you do not wish to do this, or you still have concerns about doing so, you may contact the UCT Human Research Ethics Committee using the contacts below:  **HREC Ethics Office:**  **Email:** [**hrec-enquiries@uct.ac.za**](mailto:hrec-enquiries@uct.ac.za)  **Tel: +27 21 406 6338**  The study has been explained to me in a language that I understand. All the questions I had about the study have been answered. I understand what will happen during the interviews and what is expected of me.   - I have been informed that it is my right to refuse to take part in the interviews and that if I choose to refuse I do not have to give a reason, and that my employer will not be informed now, or in the future. - I have been informed that anything I say during the interviews will be kept confidential: my name will not be used and no quotes or other results arising from my participation in this study will be included in any reports, even anonymously, without my agreement.   Circle response:   \| I agree to take part in the study: \| Yes \| No \| \| --- \| --- \| --- \|   **Signature of participant:**   \| **NAME**  (in capital letters) \| **SIGNATURE** \| **DATE OF SIGNATURE**  (in DD/MM/YYYY) \| \| --- \| --- \| --- \| \|  \|  \|  \|   **Signature of study staff taking consent:**   \| **NAME**  (in capital letters) \| **SIGNATURE** \| **DATE OF SIGNATURE**  (in DD/MM/YYYY) \| \| --- \| --- \| --- \| \|  \|  \|  \| |

| **Tool S2c** |
| --- |
| **INFORMATION AND CONSENT FORM – SEMI-STRUCTURED INTERVIEWS (IDI)**  **Smart surveillance towards malaria elimination in Mpumalanga, South Africa (SS4ME): novel approaches for mapping antimalarial resistance**  *Sub-study: Development and optimisation of Plasmodium falciparum artemisinin and partner drugs resistance maps for communication with policymakers in Southern Africa and South-East Asia* |
| **Introduction**  As you may know, we have been conducting a study to plan for a new approach for linking antimalarial drug resistance data with malaria notification data (Smart Surveillance for Malaria Elimination, SS4ME), which we piloted in Mpumalanga. We are interested in learning about how this project has been experienced from the perspective of those involved in malaria notification activities and those who would use the data generated to make policy decisions in this region. will also present some drafted maps obtained from the collected data and ask for your advice in ways we can improve them.  **Why was this study being done?**  We wanted to know how best activities related to SS4ME could be planned and integrated into the current malaria notification system. This involved the malaria elimination programme (MEP) collecting all positive RDT tests (with filter papers used to dab any blood remaining after the fingerprick) and sending them to the NICD laboratory for assays of molecular markers of resistance to Coartem®. These results were then linked to those of the malaria notifications and displayed on a map to indicate risk of antimalarial drug resistance and level of uncertainty. The results of this project will help us understand how best this SS4ME could be designed and implemented within malaria endemic areas of South Africa and potentially regionally.  **What will happen today if I take part in this study?**  Today, we would like to ask you questions about your perspective on the project we have now completed, including the value of linking these data with drug resistance marker data and how best this could be planned for and implemented now that we have completed the pilot; what worked, what didn’t work and how SS4ME may be improved. As before, we will take notes of the discussions and a recording will also be made using a digital voice recorder.  **What are my rights if I take part in this interview?**  Taking part in this interview is entirely your choice. You may choose either to take part or not to take part. If you decide to take part, you can decide to stop participating at any time. Just tell the project researcher right away if you wish to stop an interview. No matter what decision you take, there will be no penalty to you in any way.  **How long will the interview last and what risks can I expect from taking part?**  Each interview will last about 60-90 minutes. Participation in any research study may involve a loss of privacy. Information you provide about your experiences and opinions will be recorded, but your name will not be used in any reports of the information provided. No quotes or other results arising from your participation in this study will be included in any reports, even anonymously, without your agreement. The information obtained from these interviews will only be used by the project researchers and will be locked at our project offices. We will do our best to make sure that the personal information gathered for this survey is kept private.  **Are there benefits to taking part in the study?**  There will be no direct benefit to you from participating in this study. However, the information that you provide will help researchers and policy-makers understand how best to implement SS4ME.  **What are the costs of taking part in this study? Will I be paid for taking part in this study?**  There are no costs to you for taking part in this study. You will not be paid for taking part.  **Who can answer my questions about the study?**  You can talk to the researchers about any questions or concerns you have about this interview. If you have any questions, comments or concerns about taking part in this study, first talk to the researchers [Contact [name, number of project team member(s)]. If for any reason you do not wish to do this, or you still have concerns about doing so, you may contact the UCT Human Research Ethics Committee using the contacts below:  **HREC Ethics Office:**  **Email:** [**hrec-enquiries@uct.ac.za**](mailto:hrec-enquiries@uct.ac.za)  **Tel: +27 21 406 6338**  The study has been explained to me in a language that I understand. All the questions I had about the study have been answered. I understand what will happen during the interviews and what is expected of me.   - I have been informed that it is my right to refuse to take part in the interviews and that if I choose to refuse I do not have to give a reason, and that my employer will not be informed now, or in the future. - I have been informed that anything I say during the interviews will be kept confidential: my name will not be used and no quotes or other results arising from my participation in this study will be included in any reports, even anonymously, without my agreement.   Circle response:   \| I agree to take part in the study: \| Yes \| No \| \| --- \| --- \| --- \|   **Signature of participant:**   \| **NAME**  (in capital letters) \| **SIGNATURE** \| **DATE OF SIGNATURE**  (in DD/MM/YYYY) \| \| --- \| --- \| --- \| \|  \|  \|  \|   **Signature of study staff taking consent:**   \| **NAME**  (in capital letters) \| **SIGNATURE** \| **DATE OF SIGNATURE**  (in DD/MM/YYYY) \| \| --- \| --- \| --- \| \|  \|  \|  \| |
