# Supplementary material for: Factors affecting integration of an early warning system for antimalarial drug resistance within a routine surveillance system in a pre-elimination setting in Sub-Saharan Africa
Source: PLoS One. 2025 Jun 3;20(6):e0305885. doi: 10.1371/journal.pone.0305885 (PMC12132925; doi:10.1371/journal.pone.0305885)
Supplement: S3 Tool — (DOCX) [file pone.0305885.s006.docx]

| **Tool S3a: SS4ME guide for Focus Group Discussions** |
| --- |
| *A semi-structured guide for focus group discussions on integrating molecular surveillance for markers of antimalarial resistance into the routine malaria notification system* |
| \| **FGD IDNO** \|__\|__\|__\|__\| **Facilitator Initials** \|__\|__\|__\| **Note-taker Initials** \|__\|__\|__\|  **Audio file**: \|__\|__\|__\| **Date** \|__\|__/__\|__/__\|__\|  **Introduction**  I am ______________________________ from ______________________ (facilitator)  I am ______________________________ from ______________________ (note-taker)   - Ask group to introduce themselves using first names - Capture demographic details – using first name for discussion - Explain general purpose of the FGDs:   To understand the experiences of participants with malaria diagnosis, treatment and notification and their ideas about a new system   - Aims of the discussion and expected duration (1 hour) - Who is involved in the process (other participants) - Why the participants’ cooperation is important - What will happen with the collected information and how the participant/target group will benefit - Ask group to define their own ground rules, for example: - Only one person talks at a time. - It is important for us to hear everyone’s ideas and opinions. There are no right or wrong answers to questions – just ideas, experiences and opinions, which are all valuable. - It is important for us to hear all sides of an issue – the positive and the negative. - Confidentiality is assured. “What is shared in the room stays in the room.” - Any questions? - Check position and functioning of tape recorder - Check for everyone’s consent to participate and be recorded - Refreshments will be served after the discussion \| \| \| --- \| --- \| \| **Domain** \| **Topic and probes** \| \| **Roles** \| As a reminder, briefly, what does your job involve? Why is this important? \| \| **Adding resistance data to notifications** \| There are parts of the world where malaria parasites have developed resistance to all treatments available. So far the treatments recommended here still seem to work very well, but we need to improve our surveillance systems to make sure we detect and contain any drug resistance that may occur here.  The SS4ME project involved linking malaria notifications with molecular markers of antimalarial drug resistance obtained from RDT samples and blood spots sent to the NICD. We then can put the results on maps with GIS coordinates of where the RDTs were taken, where people live and sometimes where people had travelled from (if different). In time this will allow us to predict areas of drug resistant parasites which may guide where more work is needed to contain resistance and, for instance, change treatment policy.  Let’s talk about your experience of this project. (Probe: were you aware of the project? What role did you play?)  We would first like your advice on whether we need to refine the practical processes   - For instance, we asked [you][clinic staff] to label used RDTs and blood spots on filter paper to dab blood remaining after finger prick. These were collected and stored at clinics for despatch to the NICD   - What was your experience with this, if at all? Probe: labelling, bagging, labelling bags, logs, transfer to NICD (what will happen if the project coordinator is not available to help with some aspects of this, e.g. logs?)   - We then linked the malaria notifications for each patient with his or her positive RDT resistance data results from the NICD by using the Notifiable Medical Conditioner Identifier (NMC ID)   - What do you think of this? Probe: are there other identifiers that could be used instead or as well?   - What other systems have been in place for malaria notifications in the past 6 months in your facility/area? Probe: MalariaConnect®, NMC App. How was your work affected by these systems? Duplication, missing data?   During the project in the same area (Nkomazi sub-district), the department of health introduced a single low dose of a new (unregistered) drug called primaquine to reduce the transmission of malaria within the community and therefore increase the chance of malaria elimination. Clinics were asked to dose patients with uncomplicated malaria (with a few restrictions) with PQ and add a record of this dosing to the notification forms [and for case investigations an additional page 3 to assess treatment response]   - How did this impact you in your work? Probe: malaria notifications/ case investigations \| \| **Maps (MEP staff only – show maps)** \| Parasite resistance to malaria treatment has spread in eight countries in Asia and in the past resistance has previously transferred from Asia to Africa.   - Do you think we should be concerned? Why? Probe: if no, what level of resistance would give rise to concern?   As part of monitoring and early warning systems, maps of antimalarial resistance are being used to monitor spread.   - How useful are maps for monitoring drug resistance? - What type of maps, and information in them, would you prefer? Probe use of different colours for cases, resistance etc.   Here is a set of drug resistance maps produced from the Nkomazi linked data;   - Which of the formats do you find easier to understand? Why? - Would you like any changes on this format to improve it? Probe: change of colours, symbols, titles, captions, or administrative units/localities   Here is another set of maps produced from another area in the world   - If this was South Africa, what is your impression of the data? Probe: what steps might be needed on seeing these data? \| \| **Overall utility and impact** \| - What do you think are the benefits of SS4ME? And the challenges? - What do you think are the benefits of introducing SLD PQ? And the challenges? - Is this project of linking notifications and drug resistance data worthwhile to your work? Probe: can you see any value for your patients? [MEP programme - value for identifying foci for investigation] - How best can this model be scaled up? Probe: if we were to introduce this to Bushbuckridge or Mbombela, what would you advise? - MEP staff only: do you think the SS4ME project is the right one to generate information that will help you and others to do your role? \| \| **Closing**  We are now approaching the end of our discussion. Is there anything else anyone would like to add?   - Summarise - Thank participants   Collect participant demographic details \| \| |

| Tool S3b: SS4ME guide for In-depth Interviews |
| --- |
| *A semi-structured guide for in-depth interviews on integrating molecular surveillance for markers of antimalarial resistance into the routine malaria notification system*. |
| \| **IDI IDNO** \|__\|__\|__\|__\| **Facilitator Initials** \|__\|__\|__\| **Note-taker Initials** \|__\|__\|__\|  **Audio file**: \|__\|__\|__\| **Date** \|__\|__/__\|__/__\|__\|  **Introduction**  I am ______________________________ from ______________________   - General purpose of the study - Aims of the interview and expected duration - Who is involved in the process (other participants) - Why the participant’s cooperation is important - What will happen with the collected information and how the participant/target group will benefit - Any questions? - Consent   **Warm up [demographic & work history]**  Can I ask some details about you and your job?  Job title ____________________________  Years worked in this role \|__\|__\|yrs\|__\|__\|mths  Highest educational grade attained ___ __ Year of graduation____________  Are you originally from this area? □ Yes □ No \| \| \| --- \| --- \| \| **Domain** \| **Topic and probes** \| \| **Role** \| As a reminder, briefly, what does your job involve? Why is this important? \| \| **Linking resistance data to notifications** \| There are parts of the world where malaria parasites have developed resistance to all treatments currently available. So far treatments recommended here still seem to work very well, but we need to improve our surveillance systems to make sure we can promptly detect and contain any drug resistance that may occur here.  The SS4ME project involved linking data from malaria notifications with molecular markers of antimalarial drug resistance from RDT samples and blood spots by the NICD. Data were then mapped according to GIS coordinates of sampling, residence (and suspected location of infection, if different). This feeds into a geo-statistical model, yielding a predictive map of estimated prevalence of drug resistant parasites showing levels of uncertainty, to inform drug resistance containment efforts and – if needed – changes in malaria treatment policy.  Let’s talk about your experience of this project. (Probe: were you aware of the project? What role did you play?)  We would first like your advice on whether we need to refine the practical processes   - For instance, we asked clinic staff to label RDTs and blood spots on filter paper to dab blood remaining after finger prick which were collected and stored at clinics for despatch to the NICD   - What was your experience with this, if at all? Probe: labelling, bagging, labelling bags, logs, transfer to NICD (what will happen if the project coordinator is not available to help with some aspects of this, such as the logs?)   - We then linked the notification data for each patient with his or her positive RDT using the Notifiable Medical Conditioner Identifier (NMC ID)   - What do you think of this? Probe: are there other identifiers that could be used instead or as well?   - What other systems are in place for malaria notifications? Probe: MalariaConnect®, NMC App. How was the project affected by these systems and vice versa? Duplication, missing data? - During the project in the same area (Nkomazi sub-district), the department of health introduced a single low dose of a new (unregistered) drug called primaquine to reduce the transmission of malaria within the community and therefore increase the chance of malaria elimination. This involved reporting details of patients treated with PQ (notification forms) and for case investigations an additional page 3 to assess treatment response.   - How do you think this impacted the project and the routine malaria case management/case investigation system? Probes: how did introducing PQ affect notifications? How did introducing PQ affect case investigations? \| \| **Maps – show maps** \| Resistance to artemisinins has spread in eight countries in Asia. Drug resistance has previously transferred from Asia to Africa, and malaria partner drugs (like lumefantrine) have already started showing tolerance in Africa.   - Do you think we should be concerned? Why? Probe: if no, what level of resistance would give rise to concern?   As part of monitoring and early warning systems, maps of antimalarial resistance showing the distribution of drug resistance are being used to monitor geographical spread of artemisinin and lumefantrine resistance.   - How useful are maps for monitoring drug resistance? - What type of maps, and information in them, would you prefer? - [general discussion on colours for cases, resistance etc.]   Here is a set of drug resistance maps produced from the Nkomazi linked data;   - Which of the formats do you find easier to understand? Why? - Would you like any changes on this format to improve it? Probe: change of colours, symbols, titles, captions, or administrative units/localities   Here is another set of maps produced from another area in the world   - If this was South Africa, what is your impression of the data? Probe: what steps might be needed on seeing these data? \| \| **Overall utility and impact** \| Overall, do you think the SS4ME project is fit-for-purpose to generate information that will help you and others to do your role?   - What were the benefits of SS4ME? And the challenges? - What were the benefits of introducing SLD PQ? And the challenges? - Are the outputs worthwhile? Probe: opportunity costs, for identifying foci for investigation - How sustainable is this project? Probe: how best can this model be scaled up - Overall, what is the utility of this model for supporting changes to malaria treatment policy \| \| **Closing**  We are now approaching the end of our discussion. Is there anything else you would like to add?   - Summarise - Thank participant \| \| |
