# Supplementary material for: Factors affecting integration of an early warning system for antimalarial drug resistance within a routine surveillance system in a pre-elimination setting in Sub-Saharan Africa
Source: PLoS One. 2025 Jun 3;20(6):e0305885. doi: 10.1371/journal.pone.0305885 (PMC12132925; doi:10.1371/journal.pone.0305885)
Supplement: S1 Table — (DOCX) [file pone.0305885.s007.docx]

| **Table S1a: Illustration of the process-oriented logic framework without data.** |
| --- |
| *Below are table structured summaries displaying the process-oriented logic framework, including processes, inputs, outputs, and outcomes for evaluating the integration of molecular markers of resistance into a routine malaria notification system.* |
| \|  \|  \| **Logic** \| \| \| \| \| \| \|  \| \| --- \| --- \| --- \| --- \| --- \| --- \| --- \| --- \| --- \| --- \| \|  \|  \| ***Inputs/Activities*** \| \| ***Outputs*** \| \| ***Outcomes*** \| \| \|  \| \|  \|  \|  \| Intended \| \| Unintended \| Unintended \| Short-term \| Long-term \| Quotes/example \| \| Processes \| Process I, II, III … \| Activity 1, 2, 3 … \|  \| \|  \|  \|  \|  \| Quote 1, 2, 3 \| \| Process I, II, III … \| Activity 1, 2, 3 … \|  \| \|  \|  \|  \|  \| Quote 1, 2, 3 \| \| Process I, II, III … \| Activity 1, 2, 3 … \|  \| \|  \|  \|  \|  \| Quote 1, 2, 3 \| |
| **Table S1b: The process-oriented logic framework with examples of processes, inputs, outputs and selected quotes.** |

| \|  \| \| **Processes** \| \| \| \| \| \| \| --- \| --- \| --- \| --- \| --- \| --- \| --- \| --- \| \| ***Detection, notification and reporting*** \| ***Sample collection and reporting*** \| ***Case investigation*** \| ***Data capturing*** \| ***Analysis and reporting*** \| ***Others*** \| \| **Inputs/Activities** \| \| Filling forms (additional contact information and address details) \| Sample collection (dried blood spot collection and labelling) Assigning barcodes stickers \| New location (GPS) collection SoPs \| Barcode scanner Barcode scanning \| Monthly downloads of data and analysis Monthly data reporting \| Training Usage of devices Sample collection Meaning of MMR & surveillance in general \| \| **Outputs** \| *Intended* \| Completed demographics and patient tracking information \| Barcoded filter papers of blood samples for molecular marking \| Increased GPS accuracy \| Automatic capture of barcodes avoidance of transcription errors \| Data quality assessment \| Trained staff on GPS devices, DBS sample collection and filling the notification forms  Informed staff on the meaning and importance of MMR and surveillance \| \| *Unintended* \| Daunting start  Increased workload   Multiple reporting systems \| Increased workload poor packaging and filing of the sample details Lack of barcode assignment \| Extra training sessions  Sense of ownership of the project that triggered the agency  Threatened working relationships  Requirement for airtime \| Inconsistent barcode assignment  Manual input of barcodes Increased workload \| Increased workload due to the merging of the different three forms \| Increased awareness of resistance  Added confidence to staff  Positive perception of the added tasks \| \| **Outcomes** \| *Short-term* \| Resistance leads to a slow start  Incomplete tracking information \| Inadequate quality samples and data shipped to the central lab for molecular analysis \| Increased accuracy of patients' location  Motivated teams \| Non-barcoded-samples Transcription errors Slow data-capturing process \| quarterly reports of cases' notification, investigation and linkage  Feedback of areas of improvement and activities for areas of improvement \| Increased dedication to working with correct processes  Contradictions (knowing vs not knowing staff,  willing vs not-willing staff) \| \| *Long-term* \| Acceptance and adapting  Increased programmatic support and monitoring \|  \| Failure to track all patients \| Increased linked cases at the household level Overall increase in willingness and uptake of the programme \| Improvement of data quality, timeliness in reporting and willingness to carry out MMR-RMSS  Increased linkage \| Raised knowledge about malaria and drug resistance  Raised quality of data and samples.   Increased linkability \| \| **Quotes** \| \| ‘We do the paper first, the notification book first, the remaining depends with their time or workload’. [Nurse, IDI06].  ‘But the volume you can look at the CHCs [Community Health Centres] and eight hour, there are more people coming in because day and night we are working.’ [Nurse FGD03].  ‘Basically at first it was quite a daunting task because now [I] remember we had facilities that recently introduced the new NMC form and also Malaria Connect and all of that. So when we went to train the health facilities they were resistant because they feel that it is extra work on their behalf. … Then even though they complied, we were getting a hint that they were not completely filling incorrectly the samples that we needed them to.’ [Environmental Health Officer, FGD01]. \| ‘Yes, there is not enough (blood from one prick). There is a little but it is not enough. So most of the time they have to prick maybe twice or three times sometimes.’ And added by another staff member ‘I think the experience has taught us that mostly when the people are working in the farms to draw blood, sometimes they prick harder. So it is either they have to prick maybe two times or three times.’ [Case investigator, FGD01].  ‘These activities have helped us to know what we are doing and   to help the patients, if they ask me now why we are taking another blood sample, it is for monitoring drug resistance and make sure you are cured. So it’s a very good thing’ [Nurse, FGD04].  ‘remember we went with the supervisor to a couple of clinics when we identified that problem (sample lacking respective DBS or with insufficient DBS) and then we went back and told them that now we no longer just take the RDT. We also need to take the dry blood spots, so we need enough blood for the dry blood because sometimes we find that the dry blood is just a tiny bit of the blood and it really cannot be used. So we had to make sure that we go back to them and tell and train them that when you prick make sure you prick enough just in case it is a positive you can also get more blood. They are currently a lot better’ [Nurse, FGD03]. \| ‘We drive almost 40 kms or more to find a case, then the number is wrong and there is only one line for address. Just looking for one case you take the whole day while other notifications are waiting.’ [Case investigator, FGD01]  ‘Sometimes I call a clinic before leaving home to know if there are cases near where I leave. And..travelling to Clinic easy closer to where I leave than the office, so I don’t spend too much time on the road going back and forth’ [Case investigation officer, FGD05].  ‘Sometimes I call a clinic before leaving home to know if there are cases near where I leave. And..travelling to Clinic easy closer to where I leave than the office, so I don’t spend too much time on the road going back and forth’ [Case investigation officer, FGD05].  ‘Yes, they do so (call) because in the notification book we also write our phone numbers when we notify a patient, if there is something missing they can call. Even if we are not on duty, yah, threatening our working relationship.’ [Nurse, FGD03]  ‘The ownership thing you know when you understand exactly what it is you are capturing and how important it is then it makes all the difference in terms of making sure it is on file because, how these things usually get presented it is a study and there is a lot of studies that we come across and it is always just another study that we do not know how it is going to end up, who it is going to benefit. So I think also that approach of you [referring to the study team] is excellent as we know what this is.. and what this is for. This is how important it is, it is ours only. [Information officer, FG02] \| ‘When it is the rainy days and the cases increase, sometimes it’s only two of us capturing data. Therefore, we would be late to capture… Late for a few days to one or two weeks.’ [Data clerk, FGD01].  ‘Typing is easier than using a barcode scanner’. [Data clerk, FGD01].  ‘Yes, that are missing, maybe the facility code or anything like that then maybe three or four times we have experience that the RDT came alone like just the RDT without the filter paper, without a barcode. So most of the times we just discard because there is nothing we can do or go back and find this person and do the thing again’ [Surveillance supervisor, FGD02] \| ‘…what I do is to, I need to merge the NMC form together with the page three case investigation form and to take it outside the computer and merge even if it is excel it is prone to changing formats and that could introduce issues that could lead to un-matching within the DHIS2 framework.’ [Information officer, KII03].  ‘Once notifications were submitted and paper forms were captured in the HMIS system, ideally, all cases were merged and duplicates removed. In our analysis and feedback some cases had duplicates that required further cleaning.’ [Information officer, KII03].  ‘Ja I think another thing that is good our case investigation has also improved because I remember when we started our base line was thirty five percent. For forty eight hours and seventy two hours[time between case reporting and investigation] it was around forty eight percent. So now on third quarter we reported above sixty five which was even above the target we set for this financial year. I think with them making the follow ups, having to go to the facilities to check on the stocks and everything has made them to also be involved in going there and investigating the cases because the thing was XXXX and XXXX [mentioning MMR-RMSS project staff] would be analysing and supporting with feedback e.g., saying no but the Primaquine the facility is this much and then we are not having the cases recorded. So ja I think our system improved with the Primaquine and the smart surveillance it has pushed them to go and investigate the cases within the required time.’ [Supervisory staff, KII02] \| ‘The ownership thing you know when you understand exactly what it is you are capturing and how important it is then it makes all the difference in terms of making sure it is on file because, how these things usually get presented it is a study and there is a lot of studies that we come across and it is always just another study that we do not know how it is going to end up, who it is going to benefit. So I think also that approach of you [referring to the study team] is excellent as we know what this is.. and what this is for. This is how important it is, it is ours only. [Information officer, FG02] \| |
| --- | --- | --- | --- | --- | --- | --- | --- | --- | --- | --- | --- | --- | --- | --- | --- | --- | --- | --- | --- | --- | --- | --- | --- | --- | --- | --- | --- | --- | --- | --- | --- | --- | --- | --- | --- | --- | --- | --- | --- | --- | --- | --- | --- | --- | --- | --- | --- | --- | --- | --- | --- | --- | --- | --- | --- | --- | --- | --- | --- | --- |
