# Supplementary material for: Factors affecting integration of an early warning system for antimalarial drug resistance within a routine surveillance system in a pre-elimination setting in Sub-Saharan Africa
Source: PLoS One. 2025 Jun 3;20(6):e0305885. doi: 10.1371/journal.pone.0305885 (PMC12132925; doi:10.1371/journal.pone.0305885)
Supplement: Attachment — Submitted filename: Editor in chief - PONE-D24-18820 reviewers comments.pdf [file pone.0305885.s008.pdf]

Editor in chief,

I had the pleasure of reviewing the manuscript entitled *''Factors Affecting Integration of an Early Warning System for Antimalarial Drug Resistance within a Routine Surveillance System in a Pre-elimination Setting in Sub- Saharan Africa''* assigned manuscript number PONE-D-24-18820.

The manuscript is well articulated and comes at an appropriate time as many regions that were previously malaria-endemic are now moving toward pre-elimination phase. The manuscript highlights the importance of continued and sustained molecular surveillance as part of pre-elimination phase. It also identifies barriers to successful implementation and discussed potential challenges. However, I have several minor comments as indicated below.

<u>Abstract</u>

The abstract is well written and accurately links the research questions with the anticipated outcome. However,

1. The conclusion that reads *'' However, cross-cutting barriers inherent in the healthcare system can determine its success in a resource-limited setting''*. The message here is not clear. The author should consider using a different word like *''influence or affect'',* unless a different clarification of what the authors wish to convey to the reader is given.

<u>Authors</u>

2. Please use consistent formatting while writing authors with initials. For example, use John I. Smith instead of switching between ''John I Smith''. The authors need

to maintain consistency. Specifically, Frank M. Kagoro vs Richard J Maude. Please add a full stop after the initial.

Introduction

The introduction is well presented, indicating the existing gaps as well as the rationale for conducting the study.

Materials and Methods

The materials and methods section is well presented. However, there are a few areas that need improvement as indicated below:

3. The authors indicate that ''falciparum-specific histidine-rich protein 2 (HRP2)-based mRDT'' was used. HRP2 mRDTs are only relevant to *P. falciparum* mono-infection, not mixed infections that need Pf/PAN mRDTs. The authors need to clarify why HRP2 mRDTs were preferred instead of combo mRDTs that would detect mixed infection or mono-infection of species other than *P. falciparum*. Species other than *P. falciparum* could also be circulating in the area and may be missed by the HRP2-only mRDTs. If the preference for HRP2 was due to the predominance of *P. falciparum*, this should be stated somewhere to justify this choice. Otherwise indicate this as one of the study limitations.

4. Furthermore, how was the issue of HRP2 deletion managed? Were there instances where some cases were mRDT- negative but slide or PCR positive? How were such situations handled? What was the gold standard of confirmatory detection tool that was used to ascertain mRDT results?

5. It is further indicated in the methods section that *'' For molecular surveillance, DBS filter papers were to be collected from all patients with positive mRDTs''.* How were false negative cases determined, particularly in situations where only HRP2 mRDT was used, potentially missing other infections, such as *P. ovale, P. malariae, P.*

vivax and others? There is likelihood that a significant number of positive cases were missed.

6. Please also indicate the brand name and manufacturer of the mRDT used, as this is a standard practice when writing a manuscript.

7. In the main study, how was a malaria case defined? Using only mRDT seems inadequate. Were other tests such as malaria microscopy or species diagnostic PCR performed? If not what were the reasons for not doing so, and how did you differentiate mixed infections from Pf mono-infection or infections other than Pf given the fact that only HRP2 mRDT was used?

Results

8. Of the notified cases, 55.1% (n=2,636) were linked to their *Plasmodium falciparum* molecular marker resistance profiles, with 85% (n=2,240)…. Here around 45% of the cases could not be linked with the Pf molecular marker profile. This could either be due to a large number of false positive cases by mRDT that did not generate any parasite DNA for genotyping or only 55% were purposively selected for PCR analysis. The author should clarify this by at least providing a CONSORT diagram indicating how the sampling was done while depicting samples for PCR analysis.
9. The authors should also indicate whether species diagnostic PCR was performed to rule out the presence of other species or mixed infection. Figure 1 is not very elaborate on this.

10. Lines 264-265 '*'However, in responding to the survey question on which systems are used frequently for notification, 61/64 participants reported using these interchangeably: Malaria Connect (36, 59%), NMC Notification Book (15, 25%) and NMC Mobile Phone Application (3, 5%*).''

What is indicated in the parentheses needs to be specified, or this should be written as ‘*’36 (59%)”*, as it can confuse the reader. In the text it is written as ‘’five (8%)’’ which could be presented as (five, 8%). Please consider revising this part to be more concise and clearer.

11. Line 426 ‘’ *Yes, that are missing, maybe the facility code or anything like that, then maybe three or four times we have experience that the RDT [referring to mRDT] came alone like just the RDT without the’’.* Please correct the tense! Use ‘’experienced’’

12. Line 436 ‘’ *Routinely, the NMP collates and analysis malaria data at the provincial level each month and shares the*’’. Please change the word analysis to ‘’analyses’’. Also, check the font size.

13. Figure 1: Please clarify the discrepancies observed between notified, investigated and linked cases. Specifically, explain the variability between 4787 vs 3758 and 2636. What were the methodological issues related to such variation? This needs to be well defined.

14. Figure 2: The visibility is blurry. Consider redrawing or reducing the stretching.
