# Supplementary material for: Factors affecting integration of an early warning system for antimalarial drug resistance within a routine surveillance system in a pre-elimination setting in Sub-Saharan Africa
Source: PLoS One. 2025 Jun 3;20(6):e0305885. doi: 10.1371/journal.pone.0305885 (PMC12132925; doi:10.1371/journal.pone.0305885)
Supplement: Attachment — Submitted filename: Reviewer 1 & 2 Responses_Clean.docx [file pone.0305885.s010.docx]

Reviewer 1:

**Abstract**

The abstract is well written and accurately links the research questions with the

anticipated outcome. However,

1. The conclusion that reads ‘’However, cross-cutting barriers inherent in the

healthcare system can determine its success in a resource-limited setting’’ . The message here is not clear. The author should consider using a different word like ‘’influence or affect’’, unless a different clarification of what the authors wish to convey to the reader is given.

**Response:** We thank the reviewer for this suggestion, we have revised the paragraph (lines 73-74) to reflect this clarity.

“However, cross-cutting barriers inherent in the healthcare system can influence its success in a resource-limited setting.”

**Authors**

2. Please use consistent formatting while writing authors with initials. For example, use John I. Smith instead of switching between ‘’John I Smith’’. The authors need to maintain consistency. Specifically, Frank M. Kagoro vs Richard J Maude. Please add a full stop after the initial.

**Response:** The consistency has been noted. We have revised the authors’ names to reflect that on lines number 8-10.

Frank M. Kagoro ^1,2,3,4,5^, Elizabeth Allen ^1,3,4^, Jaishree Raman ^6,7,8^, Aaron Mabuza ^1,3^, Ray Magagula ^9^, Gerdalize Kok ^9^, Gillian Malatje ^9^, Philippe J. Guerin ^3,4,5^, Mehul Dhorda ^2,3,4,5^, Richard J. Maude ^2,5,11,12^, Karen I. Barnes ^1,3,5^

**Materials and Methods**

The materials and methods section is well presented. However, there are a few areas that need improvement as indicated below:

3. The authors indicate that ‘’falciparum-specific histidine-rich protein 2 (HRP2)-based mRDT’’ was used. HRP2 mRDTs are only relevant to P. falciparum mono-infection, not mixed infections that need Pf/PAN mRDTs. The authors need to clarify why HRP2 mRDTs were preferred instead of combo mRDTs that would detect mixed infection or mono-infection of species other than P. falciparum. Species other than P. falciparum could also be circulating in the area and may be missed by the HRP2-only mRDTs. If the preference for HRP2 was due to the predominance of P. falciparum, this should be stated somewhere to justify this choice. Otherwise indicate this as one of the study limitations.

**Response:** We thank the reviewers for noting this. This study used methods and data from the routine malaria notification system. As per the National Guideline and the Mpumalanga Malaria Programme, the test used during the study was HRP2-based, given the predominance of *P. falciparum*. The malaria programme also offers frequent training and quality assurance measures, such as collecting an additional 10% of the negative mRDTs that are sent to the NICD for polymerase chain reaction (PCR) for quality assurance. We have revised lines 143-147 for clarity as follows:

*Individuals identified through proactive, reactive, or passive case detection were screened for malaria using a P. falciparum specific histidine-rich protein 2 (HRP2)-based mRDT (First Response™ Malaria Ag P. falciparum HRP2 Detection Rapid Card Test, Premier Medical Corporation Ltd, India) according to National Guidelines and the Mpumalanga Provincial tender process. An additional 10% of the negative mRDTs were collected and sent to the National Institute for Communicable Diseases (NICD) for quality assurance.*

4. Furthermore, how was the issue of HRP2 deletion managed? Were there instances where some cases were mRDT- negative but slide or PCR positive? How were such situations handled? What was the gold standard of confirmatory detection tool that was used to ascertain mRDT results?

**Response:** During quality assurance testing, 10% of mRDT-negative samples were re-examined, and a small proportion (2.2%) were found to be PCR-positive. This was due to low parasite loads below the detection limit of mRDT (200 parasites/µl) but detectable by PCR (20 parasites/µl). Further investigations ruled out histidine-rich protein 2 (hrp2) deletions as the cause of false negatives. These findings are detailed in the quantitative paper [1], and the discussion has been updated to reflect this (lines 562-570):

*As this study was embedded in the routine malaria notification system, diagnostics relied on the hrp2-based mRDT, which is P. falciparum specific. This may have led to non-P. falciparum malaria cases being missed. However, regular sample testing and species detection studies have shown P. falciparum to dominate in this area, where other species are rare. Moreover, quality control measures, such as collecting 10% of negative mRDTs for further monitoring, including surveillance for hrp2/3 deletions, detected only a small proportion (2.2%) of false negatives due to low parasite densities below the detection limit of mRDT (200 parasites/µl) but detectable by PCR (20 parasites/µl) [12]. Further investigations ruled out histidine-rich protein 2 (hrp2) deletions as the cause of false negative mRDTs.*

5. It is further indicated in the methods section that ‘*’For molecular surveillance, DBS filter papers were to be collected from all patients with positive mRDTs’’*. How were false negative cases determined, particularly in situations where only HRP2 mRDT was used, potentially missing other infections, such as P. ovale, P. malariae, P. vivax and others? There is likelihood that a significant number of positive cases were missed.

**Response:** As responses to questions 3 and 4 mentioned, continuous evaluation of negative samples has shown other malaria species to be rare [1]. The malaria program also provided regular training and quality assurance, including collecting 10% of negative mRDT samples for PCR testing at the NICD. Results from these quality assurance measures are promptly integrated into national guidelines and reflected in practice. This information has been included in lines 143-147 (included in response to question 3 above).

6. Please also indicate the brand name and manufacturer of the mRDT used, as this is a standard practice when writing a manuscript.

**Response:** This has been specified (lines 144-145) as: “First Response™ Malaria Ag *P. falciparum* HRP2 Detection Rapid Card Test, Premier Medical Corporation Ltd, India”.

7. In the main study, how was a malaria case defined? Using only mRDT seems inadequate. Were other tests such as malaria microscopy or species diagnostic PCR performed? If not what were the reasons for not doing so, and how did you differentiate mixed infections from Pf mono-infection or infections other than Pf given the fact that only HRP2 mRDT was used?

**Response**: As noted above, routine diagnosis in primary healthcare facilities is based on HRP2 mRDT, molecular PCR analysis was used to confirm all malaria-positive and 10% of malaria negative infections. Of the 3748 malaria-positive RDTs, 13% (n = 477) were malaria-negative by PCR, five were pure Plasmodium malaria, and four were mixed infections (*P. malariae* and *P. falciparum*). This information has been reported previously as referenced [1] and described in the discussion as noted in the response to comment 4 above.

**Results**

8. Of the notified cases, 55.1% (n=2,636) were linked to their Plasmodium falciparum molecular marker resistance profiles, with 85% (n=2,240)…. Here around 45% of the cases could not be linked with the Pf molecular marker profile. This could either be due to a large number of false positive cases by mRDT that did not generate any parasite DNA for genotyping or only 55% were purposively selected for PCR analysis. The author should clarify this by at least providing a CONSORT diagram indicating how the sampling was done while depicting samples for PCR analysis.

**Response:** We have provided a new diagram below showing malaria cases and a sample flow chart and included in supplementary documents (Fig. S3), to explain this further, and have added a new paragraph in the discussion in lines 509-514:

*As reported previously [1], 45% of malaria cases could not be linked to their molecular profiles or localities (Figure S3). Many of these individuals were migrants who could not be followed up by case investigators due to missing or inaccurate local addresses. Additional factors contributing to this non-linkage included errors in capturing residential location data, whether due to human mistakes or technical issues with devices, further complicating the reporting process.*

*Figure S3: Malaria cases detected and laboratory data linkage data flow from March 2018 to February 2020 in Nkomazi sub-district, Mpumalanga. The notifiable medical condition (NMC) forms captured the data on malaria cases and later fed into the District Health Information System II (DHIS2). Laboratory samples included malaria rapid diagnostic tests (mRDTs) with their respective filter paper-dried blood spots, which were further analysed using PCR for confirmation and species detection.*

9. The authors should also indicate whether species diagnostic PCR was performed to rule out the presence of other species or mixed infection. Figure 1 is not very elaborate on this.

**Response:** Yes, PCR-based species diagnosis was carried out, as further explained in response to question 7 above and reported previously *[1]*. We have also added these details in Fig. S3 in the supporting information as explained above in response to comment 8.

10. Lines 264-265 ‘’However, in responding to the survey question on which systems are used frequently for notification, 61/64 participants reported using these interchangeably: Malaria Connect (36, 59%), NMC Notification Book (15, 25%) and NMC Mobile Phone Application (3, 5%).’’

What is indicated in the parentheses needs to be specified, or this should be written as ‘’36 (59%)’’, as it can confuse the reader. In the text it is written as ‘’five (8%)’’ which could be presented as (five, 8%). Please consider revising this part to be more concise and clearer.

**Response:** To avoid confusion, this description has been revised to capture that in lines 269-271.

*However, in responding to the survey question on which systems are used frequently for notification, 61/64 participants reported using these interchangeably: Malaria Connect (36, 59%), NMC Notification Book (15, 25%) and NMC Mobile Phone Application (3, 5%) (Figure 3).*

11. Line 426 ‘’Yes, that are missing, maybe the facility code or anything like that, then maybe three or four times we have experience that the RDT [referring to mRDT] came alone like just the RDT without the’’. Please correct the tense! Use ‘’experienced’’

**Response:** This quote captures verbatim the words used by the respondent, without grammatical correction.

12. Line 436 ‘’Routinely, the NMP collates and analysis malaria data at the provincial level each month and shares the’’. Please change the word analysis to ‘’analyses’’. Also, check the font size.

**Response:** This has been corrected as suggetsed.

14. Figure 2: The visibility is blurry. Consider redrawing or reducing the stretching

**Response:** Thank you for this recommendation. This figure has been revised as suggested.

Reviewer 2:

3. Materials and Methods

Line 138 1) instead of :)

**Response:** This typo has been corrected (line 137).

Line 141. Be consistent with how ‘sub-district’ is written

**Response:** All mentions of sub-district now have been revised to lower case except for the beginning of the sentence or a title.

Line 262: I'm not sure about using the HCW category as an identifier in the extracted quotes. Does it add value and will it in any way draw negative attention to that group?

**Response:** We would like to retain this grouping as it explains a major cadre responsible for malaria testing and notification at primary healthcare facilities. This cadre carries agency, role, and responsibility. Since they form most the workforce and respondents to this study, we do not expect any negative attribution linked to this explanation. Furthermore, the context of these quotes is conveyed when healthcare workers are distinguished from e.g. data capturers or surveillance officers.

Line 436: Typo ‘analysis’ instead of ‘analyzes’

Response: This has been corrected to ‘analyses’.

Line 447: Correct ‘wouldn’t’ to ‘would not’

**Response:** This typo has been corrected.

References:

1. Kagoro FM, Allen E, Mabuza A, Workman L, Magagula R, et al. Making data map-worthy—enhancing routine malaria data to support surveillance and mapping of Plasmodium falciparum anti-malarial resistance in a pre-elimination sub-Saharan African setting: a molecular and spatiotemporal epidemiology study. Malar J. 2022 Dec 1;21(1):1–19.
