# Supplementary material for: Factors affecting integration of an early warning system for antimalarial drug resistance within a routine surveillance system in a pre-elimination setting in Sub-Saharan Africa
Source: PLoS One. 2025 Jun 3;20(6):e0305885. doi: 10.1371/journal.pone.0305885 (PMC12132925; doi:10.1371/journal.pone.0305885)
Supplement: Attachment — Submitted filename: Comments_JJj.docx [file pone.0305885.s011.docx]

**Comments on the Manuscript**

Title: Factors Affecting Integration of an Early Warning System for Antimalarial Drug Resistance within a Routine Surveillance System in a Pre-elimination Setting in Sub-Saharan Africa

The manuscript presented by Karen I Barnes et al. is well-written, engaging, and provides valuable insights to the malaria research community. Overall, I find the manuscript to be of high quality; however, I have a few minor issues that need addressing or clarification. There are no major concerns.

**Minor Issues**

**Methods Section**

1. Tool Development and Validation: Please provide a clearer description of how the tools (Tool S1, S2, etc.) were developed and validated for this specific study context. Including the criteria or methods used for validation would enhance understanding.
2. Tool References (Lines 164 and 171): When referencing tools (S1, S2, etc.), I recommend adding a brief summary after each reference (e.g., “staff survey tool”) to improve readability and context for the reader.
3. Timeframes (Line 170): Instead of “3-6 June 2020,” consider rephrasing to “between 3rd and 6th June 2020” for clarity and consistency in date formatting throughout the manuscript.
4. Quantitative Analysis (Lines 185 & 186): While R programming is specified for data analysis, it would be beneficial to briefly mention the statistical tests or models applied to detect spatiotemporal trends and assess consistency in the data.

**Results Section**

1. Case Notifications (Lines 211–215): The section concisely provides essential statistics; however, the reference to “Further quantitative results are reported elsewhere” may leave readers curious about missing details. It would be helpful to clarify the aspects not included in this manuscript.
2. Summary of Key Findings: Please consider briefly summarizing key findings from the "elsewhere" report to provide readers with a fuller understanding of what this study covers.
3. Participant Feedback on Training (Lines 388-400): I recommend summarizing participant feedback on training more clearly. Highlighting common themes or significant outliers could provide additional depth to this section.
