# Supplementary material for: Factors affecting integration of an early warning system for antimalarial drug resistance within a routine surveillance system in a pre-elimination setting in Sub-Saharan Africa
Source: PLoS One. 2025 Jun 3;20(6):e0305885. doi: 10.1371/journal.pone.0305885 (PMC12132925; doi:10.1371/journal.pone.0305885)
Supplement: Attachment — Submitted filename: Comments_JJj_responses_KBFKKB.docx [file pone.0305885.s012.docx]

**Comments on the Manuscript**

Title: Factors Affecting Integration of an Early Warning System for Antimalarial Drug Resistance within a Routine Surveillance System in a Pre-elimination Setting in Sub-Saharan Africa

The manuscript presented by Karen I Barnes et al. is well-written, engaging, and provides valuable insights to the malaria research community. Overall, I find the manuscript to be of high quality; however, I have a few minor issues that need addressing or clarification. There are no major concerns.

Response: We thank the reviewer for acknowledging the quality of our manuscript and pointing to issues that would improve it’s quality. Below are our point by point response.

**Minor Issues**

**Methods Section**

1. Tool Development and Validation: Please provide a clearer description of how the tools (Tool S1, S2, etc.) were developed and validated for this specific study context. Including the criteria or methods used for validation would enhance understanding.

Answer: This content has now been added in the methodology section, Lines 164 – 169.

*A paper-based survey tool (Tool S1) and consent form (Tool S2) were collaboratively developed by the study team* *and was revised by the Mpumalanga Provincial Malaria Elimination Programme. Tools S3 (FGD guide) and S4 (KII guide) were similarly developed, with input from subject matter experts to ensure relevance and appropriateness. All tools were piloted and field-validated in collaboration with the malaria programme team, who tested them, provided feedback, and ensured their alignment with the study objectives before formal deployment.*

2. Tool References (Lines 164 and 171): When referencing tools (S1, S2, etc.), I recommend adding a brief summary after each reference (e.g., “staff survey tool”) to improve readability and context for the reader.

This has now been reflected in Lines 171 - 181.

*‘Tool S1 (staff survey tool) was administered between 1st March and 30th June 2020 to primary healthcare facility staff treating malaria patients to evaluate their practice, perception, and experience of the integration of molecular resistance markers into routine malaria surveillance system activities. For FGDs and IDIs, various cadres of staff involved in malaria case management at healthcare facilities and in the NMP were invited to participate by email or phone call. At least one staff member performing any malaria-related activities was invited per healthcare facility. After obtaining verbal and written consent using Tools S2 (b) and (c), FGDs and IDIs were conducted in English from 3rd to 6th June 2020 using pre-prepared interview FGD and KII guides (Tools S3 and S4 respectively) to maintain consistency and quality. The audio recordings were securely stored on the password-controlled study computer and later transcribed. Two study investigators listened to audio recordings with reference to the transcribed scripts and resolved any interpretation conflicts or transcription errors by consensus.’*

3. Timeframes (Line 170): Instead of “3-6 June 2020,” consider rephrasing to “between 3rd and 6th June 2020” for clarity and consistency in date formatting throughout the manuscript.

This has now been reflected and all dates in the manuscript use this format.

4. Quantitative Analysis (Lines 185 & 186): While R programming is specified for data analysis, it would be beneficial to briefly mention the statistical tests or models applied to detect spatiotemporal trends and assess consistency in the data.

We have revised this part to explain the type of analysis reported in a separate published article in Lines 192 - 195.

*‘All quantitative data analyses were conducted using R programming language (versions 3.6 and 4.0). Further quantitative methods used include spatial temporal, trend and usability analyses that have been explained elsewhere.[12]’*

For consistency the descriptive metrics, measurements and their justification are explicitly mentioned from lines 195 – 202.

*‘For consistency, quarterly aggregates of notified cases and investigated cases from each healthcare facility sampled were compared at three levels (healthcare facility, sub-district data capture centre and provincial DHIS2 records) and the median difference was computed. Consistency was defined as an equal number of cases being reported at different levels, with a difference of +/- 5 cases allowed to account for delayed reporting. Since a consistency benchmark for integrating molecular resistance data into malaria case data had not been described before, this was established by adapting the internal consistency benchmark proposed by the WHO Data Quality Assurance guideline.[19] Data were then explored quantitatively and qualitatively to identify possible causes of inconsistency.’*

**Results Section**

1. Case Notifications (Lines 211–215): The section concisely provides essential statistics; however, the reference to “Further quantitative results are reported elsewhere” may leave readers curious about missing details. It would be helpful to clarify the aspects not included in this manuscript.

Thank you for suggesting this improvement. This in now reflected in lines 221 – 222.

*‘Further quantitative results of spatial temporal, trends, usability and molecular markers are reported elsewhere.[12]’*

2. Summary of Key Findings: Please consider briefly summarizing key findings from the "elsewhere" report to provide readers with a fuller understanding of what this study covers.

Thank you. We have now included this summary from lines 221 to 227.

*‘No validated or associated artemisinin-resistant Kelch-13 mutations were identified among the 2,385 PCR-positive samples, while nearly all 2,812 samples analysed for lumefantrine susceptibility carried the wild-type mdr1 86ASN and crt 76LYS alleles, potentially associated with reduced lumefantrine susceptibility. Concurrently, the spatial accuracy of malaria case coordinates improved, with the average nearest neighbour distance decreasing from 330 km in the second quarter to 35 km by the fifth quarter. Further quantitative results of spatial temporal, trends, usability and molecular markers are reported elsewhere.[12]’*

3. Participant Feedback on Training (Lines 388-400): I recommend summarizing participant feedback on training more clearly. Highlighting common themes or significant outliers could provide additional depth to this section.

Response: Thank you for noting that, we have decided to reorganise this part and add depth with regards to training in lines 400 – 407

*‘Participant feedback emphasised the need for regular refresher training on malaria surveillance, particularly focusing on malaria notification and accurate case information capture at the facility level. Common themes included the importance of providing clear guidance through updated SOPs and facility guidebooks, which participants noted were instrumental in improving their confidence and efficiency. While some participants highlighted the additional workload associated with learning the new SOPs, most acknowledged the benefits, reporting improved accuracy of coordinates and overall enhancements in the NMP’s surveillance system functioning. These insights affirm the value of structured training and clear procedural support in addressing key gaps in malaria surveillance.’*
