# Supplementary material for: Factors affecting integration of an early warning system for antimalarial drug resistance within a routine surveillance system in a pre-elimination setting in Sub-Saharan Africa
Source: PLoS One. 2025 Jun 3;20(6):e0305885. doi: 10.1371/journal.pone.0305885 (PMC12132925; doi:10.1371/journal.pone.0305885)
Supplement: Attachment — Submitted filename: Responses to reviewer 1.odt [file pone.0305885.s013.odt]

Responses to Reviewer 1:
This is an interesting manuscript deserving publication in plos One. For the ease of the reader I have some suggestions to improve clarity:
Response: Thank you.
The manuscript report data from the national malaria control program, however it seems that there are only 2 authors from the provincial program included. Affiliation 10 which seems to be a combination of the national (?) malaria program and the CHAI is not indicated.
Response: We would like to point out, and as indicated in the affiliations, that co-authors 5, 6, and 7 work in the Mpumalanga Provincial Malaria Elimination Programme. Authors 2, 3, and 12 serve in the South African Malaria Elimination Committee supporting the National Department of Health.
The title suggest that this is a multi-country report across sub-Saharan Africa. It would be better for the authors to clearly identify in the title that this is a report from south Africa.
Response: The title clearly states this is a study in a pre-elimination setting in Sub-Saharan Africa and not across Sub-Saharan Africa. We would like to retain the title as it clearly states the context of the study and other contexts that would benefit from these results.
The same concern applied to the introduction (line 94-96) where it seems authors make a statement suggesting evidence derives from more than one location in South Africa.
Response: The second paragraph of the introduction unpacks the strategy for responding to antimalarial drug resistance, recommendations for integrating resistance monitoring in routine systems, and implications for pre-elimination settings. The following paragraph contextualises this information to the Nkomazi sub-district in Mpumalanga, our study area. Therefore, we find no reason for the readers to misinterpret.
Line 118-120 and remaining paragraph . Unclear how this framework looks like. No reference given. Better to provide some details for the reader to understand why it is limited. Also unclear here if you use the presented study to update and refine the framework.
Response: We have revised the manuscript from lines 119 – 122 to explain further about this framework. 
For SS4ME, a conceptual framework was developed to explore and guide how the roll-out, adoption and utilisation of new and existing technologies would inform malaria elimination goals. The SS4ME rollout would involve optimising and linking existing technologies (molecular surveillance for markers of resistance) with key functions situated at clinics, such as malaria testing, treating, referral, and notification, and district / provincial level, namely collation of malaria notification metrics and case investigation.
On lines, 125 – 127, we have explained that this study wanted to revise and build further on this framework.
Therefore, this approach needed to be revised and expanded to identify internal, external and interactive factors that could affect the implementation and impact of the intervention. 
Methods. It would be helpful for the reader to clearly differentiate between methods of the surveillance system and methods of the study presented.
Response: The introduction (lines 111 – 115) included surveillance as per the South African Malaria Guidelines. As clarified in lines 119-122 added in response to the comment, the innovation was in the linking of these routine activities. 
As per national malaria treatment guidelines, all suspected malaria cases presenting to health facilities should be confirmed by a mRDT or microscopy (passive case detection) before treatment is administered.[17]  Additionally, in pre-elimination areas of South Africa, the NMP routinely screens high-risk groups, such as migrant and mobile populations (proactive case detection), and households surrounding the residence of index cases (reactive case detection).[6,7] 
The first paragraph of the materials and methods has summarised the methods of the study followed by subsequent paragraphs explaining components of surveillance and additional assessments involved. 
Methods. Unclear based on what data the survey tool was developed. Was there any piloting done etc.
Response: All survey tools were collaboratively developed and tested in collaboration with the malaria programme. This information has been provided in lines 169 – 174.
A paper-based survey tool (Tool S1) was collaboratively developed by the study team and was revised by the Mpumalanga Malaria Programme and approved by the University of Cape Town Health Research Committee. Tools S3 (FGD guide) and S4 (KII guide) were similarly developed, with input from subject matter experts in the team to ensure relevance and appropriateness. All tools were piloted and field-validated in collaboration with the malaria programme team, who tested them, provided feedback, and ensured their alignment with the study objectives before formal deployment.
Methods: qual analysis (line 194) unclear what conceptual framework was used.
We have added further text (underlined) to make this more understandable in lines 207 – 210
For the qualitative data, each audio recording was transcribed and imported to NVivo 12 before being coded deductively, based on the conceptual framework (linking technologies, processes and key functions) and interview guides, and inductively from other observations. The first coding cycle assigned labels to text excerpts in the transcripts as lowest level ‘nodes’. 
In the method section it seems that the three elements i) the monthly and quarterly analysis, ii) the survey and iii) the qual work are all presented. But then in the results it seems that i) is reported elsewhere already. Need to make clearer in methods already. Line 231 unclear if you are referring to the quarterly assessments mentioned under i) or to the surveys? Was the survey done multiple times?
Response. All the monthly and quarterly assessments and survey and qualitative results are presented in this manuscript. Only spatial temporal analysis, trends and molecular markers are reported in the previously published manuscript. This is captured on lines 232 – 233.
Further quantitative results of spatial temporal, trends, usability and molecular markers are reported elsewhere.[12]

Suggest to structure the results better. In line 224-227 you present some emerging themes from the qual data analysis and again in line 235-237 you present additional /different themes. Unclear if they came from different analysis. Its also unclear how you defined themes and key process a priori. Perhaps it would help the reader to have a clearer understanding of your process framework in the methods section and how this fits in with your analysis framework.
Response: We have corrected a typo to clarify the key processes identified (lines 247-250).
Several key processes emerged from discussions on the integration of molecular resistance markers into the routine malaria surveillance system, which included malaria case notification, sample collection, packaging and transportation, case investigation, data capture and reporting. 
The themes and subthemes are explained, lines 258 – 261.
In Fig. 2 below, prevalent themes in the FGDs and IDIs included ease of use, perceived usefulness of surveillance, staff members’ reluctance to adopt new activities, contradictions in best practice definitions, workload, and system support. These themes converged into sub-themes: work commitment, agency and ownership, challenging processes, compromise, staffing needs, training, leadership, and supervision. 
Discussion: a detailed discussion on how the approach of focusing on the process logic is a limitation to a more holistic understanding of how people make sense of new interventions /system would be beneficial."
Response: We have added a paragraph to discuss this limitation, lines 603 - 610 .
Focusing on a process-oriented logic model can limit understanding of how individuals adapt to new interventions by emphasising operational steps over user perspectives. While effective for mapping interactions between technologies, people, and processes, it may overlook how healthcare workers and investigators interpret and adapt to molecular resistance markers in routine malaria notification within their unique sociocultural and institutional contexts. Therefore, this approach may risk neglecting critical factors like trust, resistance, and informal workflows that emerge during implementation. However, the iterative nature of this study and its focus on end-users allowed it to capture both functional factors (e.g., resistance) and relational factors (e.g., trust), addressing some of these limitations.
