# Supplementary material for: Factors affecting integration of an early warning system for antimalarial drug resistance within a routine surveillance system in a pre-elimination setting in Sub-Saharan Africa
Source: PLoS One. 2025 Jun 3;20(6):e0305885. doi: 10.1371/journal.pone.0305885 (PMC12132925; doi:10.1371/journal.pone.0305885)
Supplement: Attachment — Submitted filename: Responses to Reviewer 1-5March2025.docx [file pone.0305885.s014.docx]

Responses to Reviewer 1:

Thank you for your detailed review. We appreciate your feedback and the opportunity to clarify our use of the conceptual framework and process-oriented logic model used.

1. Clarification of the conceptual framework (SS4ME) and process-oriented logic model (Molecular Surveillance) used:

The implementation for the SS4ME study was designed to explore the roll-out, adoption, and utilization of technologies for malaria elimination. It focused on high-level determinants: problem identification, treatment-related issues, community-based interventions, outcomes, and impact. However, this implementation did not specifically account for the integration of molecular resistance markers into routine surveillance—the focus of this sub-study. To better evaluate barriers and enablers, an iterative process-oriented logic model was developed to track the roll-out of these components and assess their interactions with the surveillance system.

2. Justification for Placing the Conceptual Framework (SS4ME implementation) in the Background rather than Methods Section

The SS4ME implementation was discussed in the background to provide context for the sub-study reported in this manuscript. In order to avoid confusion and for clarity, we have revised the background from lines 117 – 119 and 123-125 to avoid reference to that conceptual framework, as stated below:

*For SS4ME, implementation focused on exploring and guiding how the roll-out, adoption and utilisation of new and existing technologies would advance malaria elimination goals.*

*As with other implementation research, this roll-out lacked a mechanism to explore the interaction between the different determinants of success and the users or beneficiaries.*

This sub-study focuses on the feasibility of linking molecular resistance markers within the malaria surveillance system, requiring a different framework tailored to assessing barriers, enablers, and implementation challenges. The methodology section has been revised to make this distinction and describe the process-oriented logic model used for this sub-study, as shown further below.

3. Reviewer's Request for a Figure

Structured summary tables of the process-oriented logic model have been included as Tables S1 – S2 to visually represent the key evaluated components and their interactions. We have revised the text in lines 207 - 209 to remove the confusion introduced by mention of the SS4ME conceptual framework and explicitly reference this figure in the methodology section:

*For the qualitative data, each audio recording was transcribed and imported to NVivo 12 before being coded deductively, based on the interview guides and the process-oriented logic model (Tables S1), and inductively from other observations.*

The process-oriented logic model was developed to systematically analyse:

- Inputs (e.g., resources, infrastructure),
- Activities (e.g., implementation steps, training),
- Outputs (e.g., data linkages, user practices),
- Outcomes (e.g., integration success, surveillance improvements).

This allowed for a more structured evaluation of implementation barriers and enablers.

We have revised to include these examples for further clarity from lines 222-228:

*As a complex intervention, the study used a process-oriented logic model to link the overall qualitative and quantitative data to determine the enablers and barriers that would explain the changing trends. To do so, results were further categorised into processes and logically analysed to identify inputs (e.g., resources, technology components) and activities (e.g., implementation steps, training), intended and unintended outputs (e.g., data linkage, use practices), and outcomes (e.g., integration success, surveillance improvements). A matrix was constructed to explore the level of influence and performance for each key feature identified from the process logic model and is further presented in the results section.*

4. References for the discussion

We have revised this section (lines 612-619) with references added for further clarity:

*Focusing on a process-oriented logic model can limit understanding of how individuals adapt to new interventions by emphasising operational steps over user perspectives. While effective for mapping interactions between technologies, people, and processes, it may overlook how healthcare workers and investigators interpret and adapt to molecular resistance markers in routine malaria notification within their unique sociocultural and institutional contexts. Therefore, this approach may risk neglecting critical factors like trust, resistance, and informal workflows that emerge during implementation.[33] However, the iterative nature of this study and its focus on end-users allowed it to capture both functional factors (e.g., resistance) and relational factors (e.g., trust), addressing some of these limitations.[34]*

We have ensured all the clarifications made are in the final manuscript. Thank you for your constructive feedback.
